# Finding enzyme cofactors in Protein Data Bank

**DOI:** 10.1093/bioinformatics/btz115

**Published:** 2019-02-13

**Authors:** Abhik Mukhopadhyay, Neera Borkakoti, Lukáš Pravda, Jonathan D Tyzack, Janet M Thornton, Sameer Velankar

**Affiliations:** European Molecular Biology Laboratory (EMBL), European Bioinformatics Institute (EMBL-EBI), Cambridge, UK

## Abstract

**Motivation:**

Cofactors are essential for many enzyme reactions. The Protein Data Bank (PDB) contains >67 000 entries containing enzyme structures, many with bound cofactor or cofactor-like molecules. This work aims to identify and categorize these small molecules in the PDB and make it easier to find them.

**Results:**

The Protein Data Bank in Europe (PDBe; pdbe.org) has implemented a pipeline to identify enzyme cofactor and cofactor-like molecules, which are now part of the PDBe weekly release process.

**Availability and implementation:**

Information is made available on the individual PDBe entry pages at pdbe.org and programmatically through the PDBe REST API (pdbe.org/api).

**Supplementary information:**

Supplementary data are available at *Bioinformatics* online.

## 1 Introduction

Almost 80% of entries (115 274 out of 144 464 as of September 2018) in the Protein Data Bank (PDB) (wwPDB Consortium, 2019) contain at least one small molecule bound to protein or nucleic acids. Presently, reasons for the presence of these molecules is not well described by the PDB annotation procedure (Young *et al*., 2018): some may be biologically relevant such as substrates, products or inhibitors, whereas others are molecules added to increase protein stability or facilitate crystallization. To identify role of these molecules we have first focussed on enzymes and their small molecule organic cofactors. The PDB has >67 000 structures of enzymes and over 30% of these enzymes require cofactors to function, making this a non-trivial problem. Relying on the knowledge of reactions catalysed by these enzymes, the classification of cofactors into 27 classes in the CoFactor database (Fischer *et al.*, 2010) and a new method to measure molecular similarity (Tyzack *et al*., 2018), we have developed a protocol to identify cofactors and cofactor-like molecules in cofactor-dependent enzymes.

The information is updated weekly with each PDB release and is stored in the PDBe database (Mir *et al*., 2018). The up to date information is made available via PDBe REST API, query system and the PDBe entry pages.

## 2 Materials and methods

### 2.1 Sources of information

The initial data were obtained from the CoFactor database (Fischer *et al.*, 2010) including manually curated information for 27 cofactor classes (Supplementary Table S1) and a list of EC numbers of cofactor-binding enzymes that are known to require each of these cofactor classes. A template molecule is defined for each of the 27 cofactor classes. The initial list of manually curated EC numbers from the CoFactor database has been expanded to include EC numbers for all non-metal cofactor-binding enzymes available in the BRENDA database (Placzek *et al*., 2017) (Supplementary Table S2). The process also uses the wwPDB chemical component dictionary (Westbrook *et al*., 2015) that contains descriptions of all unique chemical components in the PDB. The SIFTS resource (Dana *et al*., 2019) is used to obtain up to date mapping of PDB entries to enzyme commission numbers.

### 2.2 Identification and categorizing procedure

**Fig. 1.**
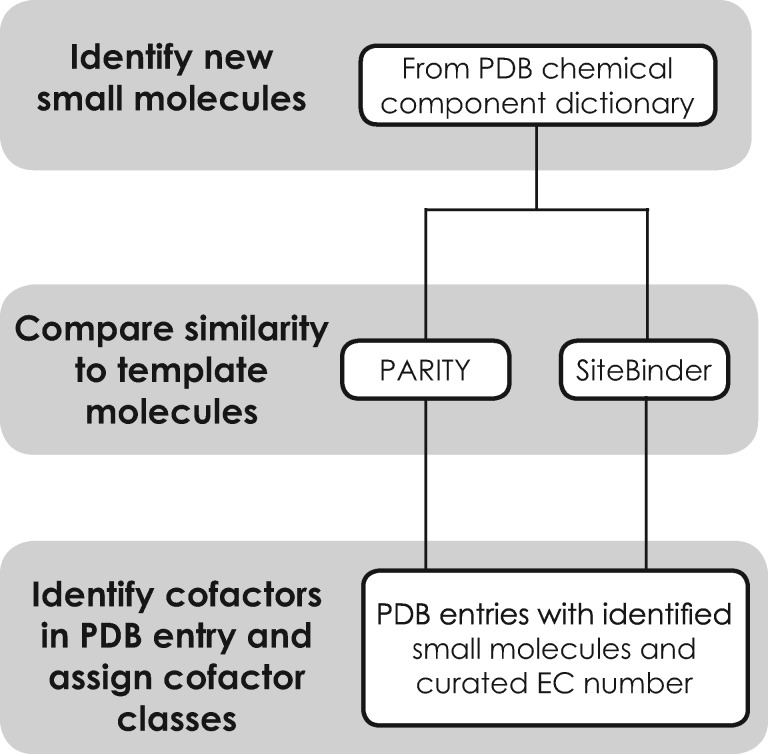
The steps implemented to identify new bound molecules that are cofactor or cofactor like

A semi-automated process (Fig. 1) has been integrated in the PDBe’s weekly release pipeline. The main steps of this procedure are

1. Newly released small molecules are identified from the chemical component dictionary. Small molecules that are structurally similar to the cofactor template molecules (Supplementary Table S3) are identified. A chemical structure similarity score is calculated using RDKit-based similarity-searching methods PARITY (Tyzack *et al*., 2018) and SiteBinder (Sehnal *et al*., 2012).
2. Small molecules with a similarity score above a predefined cut-off specified for a particular cofactor class are tentatively selected for further manual inspection for appropriate structural equivalence with the template molecule (Supplementary Table S3) and are added to the list of small molecules in the corresponding cofactor classes.
3. An automatic process obtains a list of PDB entries containing newly identified cofactor-like small molecule and enzymes associated with the corresponding cofactor classes. If the enzymes identified from the PDB entries are from the curated list of cofactor-binding enzymes, the small molecule is identified as a cofactor-like molecule in the context of the PDB entry

## 3 Results

As of September 21, 2018, we have identified 364 unique cofactor and cofactor-like molecules found in 11% of PDB entries (16 022 out of 144 464 entries). The distribution of these small molecules across enzyme classes is provided in supplementary information (Supplementary Fig. S1). All new cofactor or cofactor-like molecules and associated PDB entries are processed weekly.

### 3.1 Cofactor annotation on PDBe web pages

Cofactor and cofactor-like molecules are now clearly identified in the ligands and environments section of a PDBe entry page (Supplementary Fig. S2). Additional details on cofactor class and the similarity to the template molecule are shown on the ligand page (Supplementary Fig. S3). It is also possible to find all the cofactor-like molecules and associated PDB entries for a specific cofactor class using PDBe’s advanced search.

### 3.2 Data retrieval using the cofactor API

Three calls have been designed to retrieve cofactor data through the PDBe’s REST API (Supplementary Table S4). The most general call retrieves information of all cofactor-like molecules organized into the 27 cofactor classes along with the EC numbers of chemical reactions they catalyse. The cofactor information specific to a PDB entry can be obtained via an API call by providing the PDB entry id. For example, coenzyme A acts as a cofactor in *Clostridium acetobutylicum* thiolase (PDB id: 4xl4; pdbe.org/4xl4), but does not in pantothenate kinase 3 (PDB id: 3mk6; pdbe.org/3mk6). The last API call takes the PDB Chemical Component ID as input and lists the cofactor classes the small molecule belongs to, the cofactor class template molecule, all structurally similar cofactor-like small molecules from the identified cofactor class and their similarity to the template molecule.

## 4 Conclusion and future directions

This work provides a new way of finding cofactor related information from experimentally determined enzyme structures in the PDB. The information is also made available for integration into other biomedical data resources. Work is under way to extend the cofactor classes to include other cofactor molecules such as ATP. The details of the method and a complete analysis of the results will be presented elsewhere (in preparation).

## Supplementary Material

btz115_Supplementary_Information
