# Supplementary material for: Finding enzyme cofactors in Protein Data Bank
Source: Bioinformatics. 2019 Feb 13;35(18):3510–1. doi: 10.1093/bioinformatics/btz115 (PMC6748742; doi:10.1093/bioinformatics/btz115)

Supplementary Information

Tables

Table 1: Details of the 27 cofactor classes in the CoFactor database and the template molecule identified for each cofactor class

Cofactor class	Cofactor type	Reference chemical component ID from PDB chemical component dictionary	Chemical structure	Smiles code
1	**Thiamine diphosphate**	**TDP**	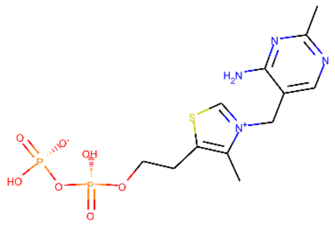	Cc1c(sc[n+]1Cc2cnc(nc2N)C)CCO[P@@](=O)(O)O[P@](=O)(O)[O-]
2	**Flavin adenine dinucleotide**	**FAD**	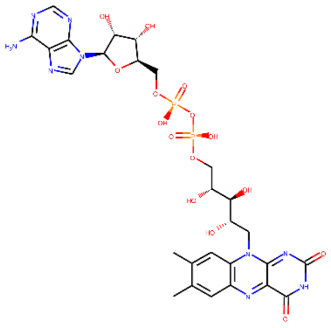	[P@](=O)(O)OC[C@@H]4[C@H]([C@H]([C@@H](O4)n5cnc6c5ncnc6N)O)O)O)O)O
3	**Flavin Mononucleotide**	**FMN**	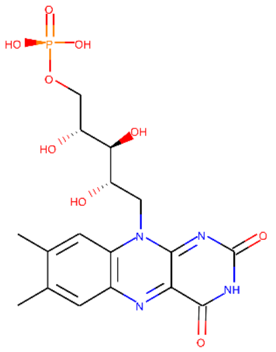	Cc1cc2c(cc1C)N(C3=NC(=O)NC(=O)C3=N2)C[C@@H]([C@@H]([C@@H](COP(=O)(O)O)O)O)O

**4**	**Nicotinamide-adenine dinucleotide**	**NAD**	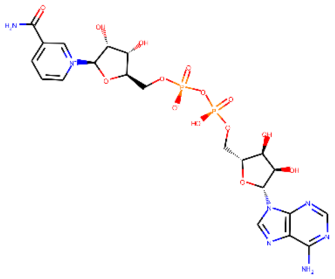	c1cc(c[n+](c1)[C@H]2[C@@H]([C@@H]([C@H](O2)CO[P@@](=O)([O-])O[P@@](=O)(O)OC[C@@H]3[C@H]([C@H]([C@@H](O3)n4cnc5c4ncnc5N)O)O)O)O)C(=O)N
**5**	**Phosphopantetheine**	**PNS**	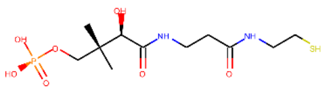	CC(C)(COP(=O)(O)O)[C@H](C(=O)NCCC(=O)NCCS)O
**6**	**Coenzyme A**	**COA**	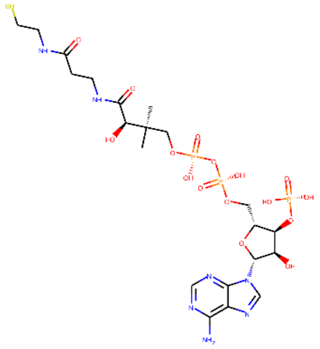	CC(C)(CO[P@](=O)(O)O[P@@](=O)(O)OC[C@@H]1[C@H]([C@H]([C@@H](O1)n2cnc3c2ncnc3N)O)OP(=O)(O)O)[C@H](C(=O)NCCC(=O)NCCS)O
**7**	**Pyridoxal 5'-phosphate**	**PLP**	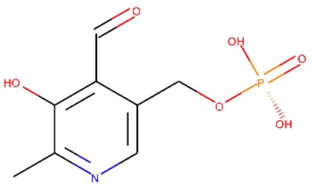	Cc1c(c(c(cn1)COP(=O)(O)O)C=O)O
**8**	**Glutathione**	**GSH**	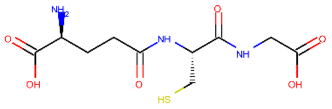	c1cc(ccc1CSC[C@@H](C(=O)NCC(=O)O)NC(=O)CC[C@@H](C(=O)O)[NH3+])

**9**	**Biotin**	**BTN**	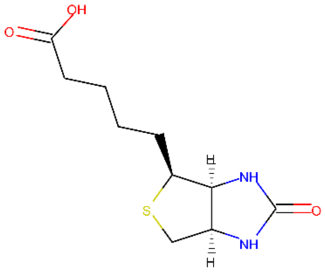	C1[C@H]2[C@@H]([C@@H](S1)CCCCC(=O)O)NC(=O)N2
**10**	**Tetrahydrofolic acid**	**FFO**	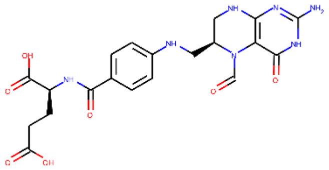	O=C(O)CC[C@@H](C(=O)O)NC(=O)c1ccc(cc1)NC[C@H](N2)CNc(c23)nc(N)[nH]c3=O
**11**	**Adenosylcobalamin**	**B12**	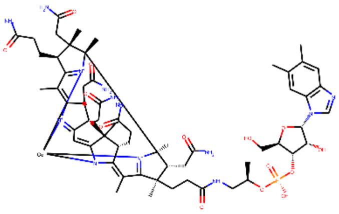	Cc1cc2c(cc1C)n(cn2)C3C(C(C(O3)CO)OP(=O)(O)OC(C)CNC(=O)CCC4(C(C5C6(C(C(C7=[N]6[Co+2]89[N]5=C4C(=C1[NH]8C(=CC2=[N]9C(=C7C)C(C2CCC(=O)N)(C)CC(=O)N)C(C1CCC(=O)N)(C)C)C)CCC(=O)N)(C)CC(=O)N)C)CC(=O)N)C)O
**12**	**Ascorbic acid**	**ASC**	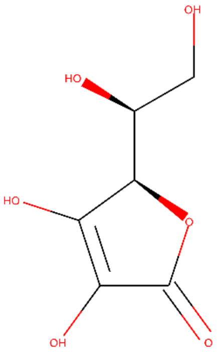	C([C@@H]([C@@H]1C(=C(C(=O)O1)O)O)O)O

**13**	**Menaquinone**	**MQ7**	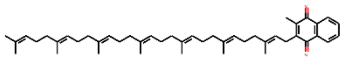	CC(C)=CCC/C(C)=C/CC/C(C)=C/CC/C(C)=C/CC/C(C)=C/CC/C(C)=C/CC/C(C)=C/CC(C1=O)=C(C)C(=O)c(c12)cccc2
**14**	**Ubiquinone**	**UQ1**	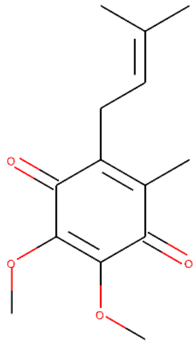	CC1=C(C(=O)C(=C(C1=O)OC)OC)CC=C(C)C
**15**	**Molybdopterin**	**MGD**	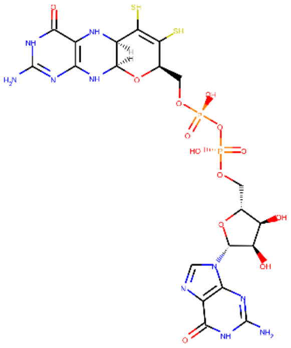	c1nc2c(n1[C@H]3[C@@H]([C@@H]([C@H](O3)CO[P@@](=O)(O)O[P@@](=O)(O)OC[C@@H]4C(=C([C@H]5[C@@H](O4)NC6=C(N5)C(=O)NC(=N6)N)S)S)O)O)N=C(NC2=O)N
**16**	**Biopterin**	**H4B**	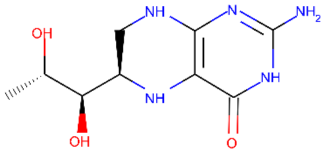	C[C@@H]([C@@H]([C@H]1CNC2=C(N1)C(=O)NC(=N2)N)O)O
**17**	**MIO (4-methylidene-imidazole-5-one)**	**MDO**	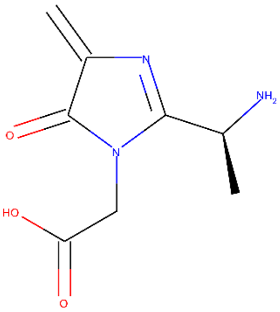	C[C@@H](C1=NC(=C)C(=O)N1CC(=O)O)N

**18**	**S-adenosylmethionine**	**SAM**	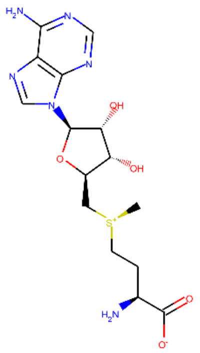	C[S@@+](CC[C@@H](C(=O)[O-])N)C[C@@H]1[C@H]([C@H]([C@@H](O1)n2cnc3c2ncnc3N)O)O
**19**	**Factor F430**	**F43**	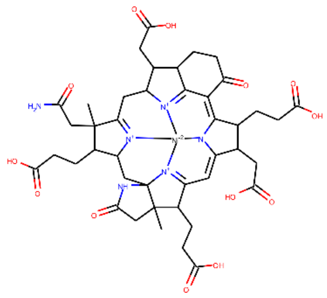	NC(=O)C[C@@]1(C)[C@H](CCC(=O)O)[C@H](C2)[N+]3=C1Cc4c(CC(=O)O)c(c5n4[Ni]36N7C8[C@@H](CCC(=O)O)[C@]([C@@]279)(C)CC(=O)N9)CCC(=O)C5=C1N6C(=C8)[C@@H](CC(=O)O)[C@@H]1CCC(=O)O
**20**	**Coenzyme M**	**COM**	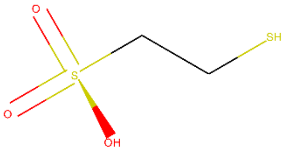	SCCS(=O)(=O)O
**21**	**Coenzyme B**	**TP7**	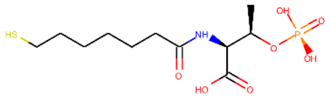	O=P(O)(O)O[C@H](C)[C@@H](C(=O)O)NC(=O)CCCCCCS
**22**	**Heme**	**HEA**	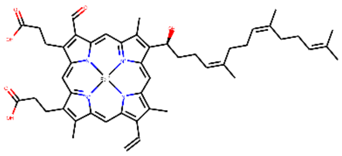	CC(C)=CCC/C(C)=C/CC/C(C)=C/CC[C@H](O)C1=C(C)C(=[N+]2C1=Cc3c(C)c(C=C)c4n3[Fe]256)C=c7n5c(c(c7C=O)CCC(=O)O)=CC8=[N+]6C(=C4)C(C)=C8CCC(=O)O

**24**	**Dipyrromethane**	**DPM**	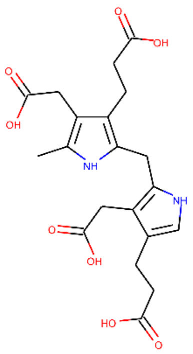	O=C(O)CCC(=C1CC(=O)O)[C@@H](N=C1C)CC2=NCC(CCC(=O)O)=C2CC(=O)O
**25**	**Pyrroloquinoline Quinone**	**PQQ**	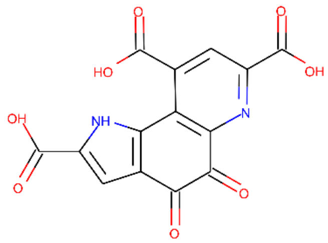	O=C(O)c(c1)[nH]c(c12)c3c(C(=O)C2=O)nc(C(=O)O)cc3C(=O)O
**26**	**Topaquinone**	**TPQ**	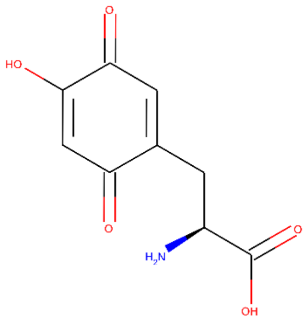	C1=C(C(=O)C=C(C1=O)O)C[C@@H](C(=O)O)N
**27**	**Orthoquinone**	**TRQ**	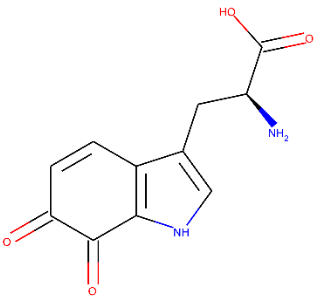	O=C(O)[C@@H](N)Cc(c[nH]1)c(c12)C=CC(=O)C2=O
**28**	**Lipoic acid**	**LPA**	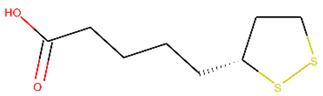	C1CSS[C@@H]1CCCCC(=O)O

Table 2. The complete set of EC numbers identified using information from CoFactor and BRENDA databases for each cofactor class

Cofactor class ID	Representative chemical component id from PDB for each Cofactor class	EC numbers for each Cofactor class from the CoFactor Database	Additional EC numbers identified for each cofactor class from the BRENDA Database	EC numbers identified for a Cofactor class
1	TDP	30	19	49
2	FAD	235	316	551
3	FMN	43	154	197
4	NAD	745	365	1110
5	PNS	8	5	13
6	COA	237	17	254
7	PLP	138	87	225
8	GSH	35	10	45
9	BTN	11	4	15
10	FFO	6	22	28
11	B12	15	33	48
12	ASC	20	16	36

**13**	**MQ7**	**1**	**10**	**11**
**14**	**UQ1**	**7**	**16**	**23**
**15**	**MGD**	**14**	**27**	**41**
**16**	**H4B**	**6**	**4**	**10**
**17**	**MDO**	**4**		**4**
**18**	**SAM**	**170**	**105**	**275**
**19**	**F43**	**1**		**1**
**20**	**COM**	**1**	**1**	**2**
**21**	**TP7**	**1**		**1**
**22**	**HEA**	**127**	**217**	**344**
**24**	**DPM**		**1**	**1**
**25**	**PQQ**	**9**		**9**
**26**	**TPQ**		**3**	**3**
**27**	**TRQ**	**1**		**1**
**28**	**LPA**	**6**	**2**	**8**
**Grand Total**		**1871**	**1434**	**3305**

Table 3: Cofactor class description, match score limits to the template and total number of chemical components

Cofactor Class ID	Cofactor class name	Cofactor representative chemical component id	Number of chemical components	Match score limit

1	Thiamine diphosphate	TDP	38	0.6
2	Flavin adenine dinucleotide	FAD	15	0.87
3	Flavin Mononucleotide	FMN	7	0.86
4	Nicotinamide-adenine dinucleotide	NAD	44	0.68
5	Phosphopantetheine	PNS	1	1
6	Coenzyme A	COA	75	0.67
7	Pyridoxal 5'-phosphate	PLP	10	0.8
8	Glutathione	GSH	53	0.42
9	Biotin	BTN	5	0.58
10	Tetrahydrofolic acid	FFO	8	0.94
11	Adenosylcobalamin	B12	4	0.84
12	Ascorbic acid	ASC	1	1
13	Menaquinone	MQ7	1	1
14	Ubiquinone	UQ1	6	0.53
15	Molybdopterin	MGD	9	0.43
16	Biopterin	H4B	8	0.78

17	MIO	MDO	1	1
18	S-adenosylmethionine	SAM	24	0.6
19	Factor F430	F43	2	0.94
20	Coenzyme M	COM	1	1
21	Coenzyme B	TP7	6	0.75
22	Heme	HEA	25	0.52
24	Dipyrromethane	DPM	3	0.97
25	Pyrroloquinoline Quinone	PQQ	1	1
26	Topaquinone	TPQ	10	0.55
27	Orthoquinone	TRQ	4	0.88
28	Lipoic acid	LPA	2	1
	Grand total		364	

Table 4: Summary of the cofactor API endpoints

Method	Endpoint	Description
GET	https://www.ebi.ac.uk/pdbe/api/pdb/compound/cofactors	Summary of the cofactor annotation in the PDBe
GET	https://www.ebi.ac.uk/pdbe/api/pdb/entry/cofactor/:pdbid	Cofactors present in a PDB entry.
GET	https://www.ebi.ac.uk/pdbe/api/pdb/compound/cofactors/het/:id	List of cofactor-like molecules similar to the query and their similarity to the template.

Figure

Figure 1 - Distribution of cofactor-like chemical components as observed in the PDB chemical component dictionary across Enzyme classes

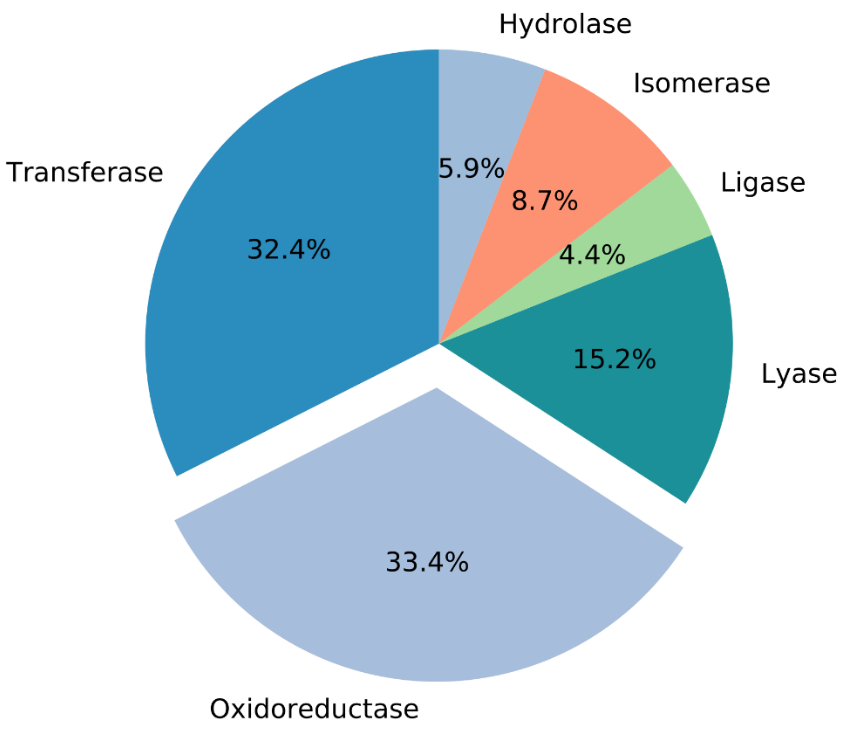

Figure 2 the PDBe entry page (pdbe.org/5hht) where the newly identified cofactor molecules are displayedunder the “Ligand and Environment” section.

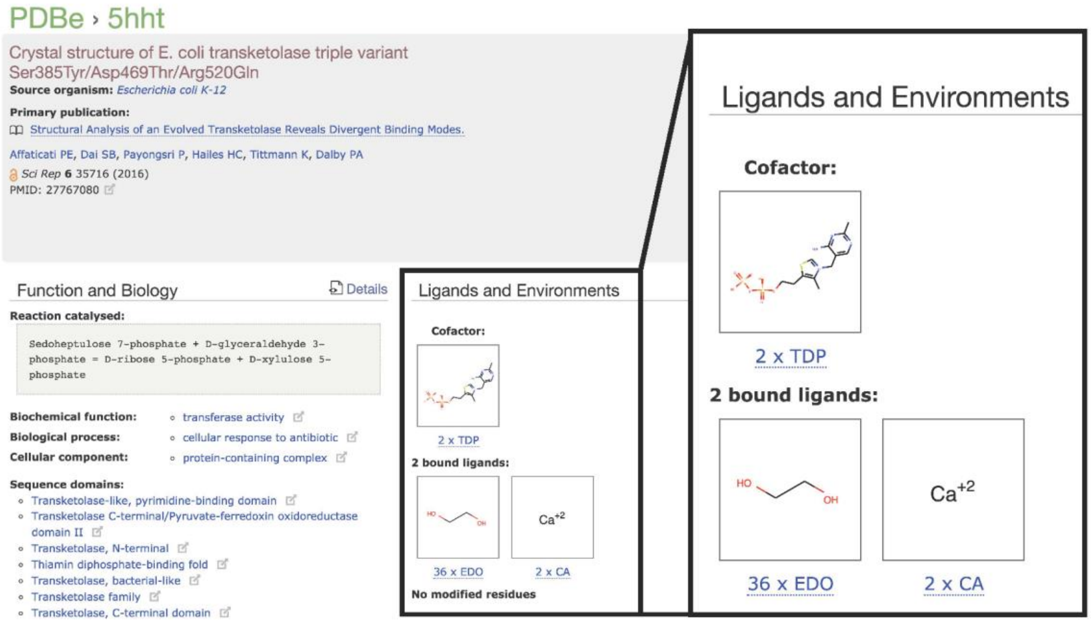

Figure 3 Cofactor class and the similarity to the template molecule are shown on the individual ligand page (http://www.ebi.ac.uk/pdbe/entry/pdb/5hht/bound/TDP).

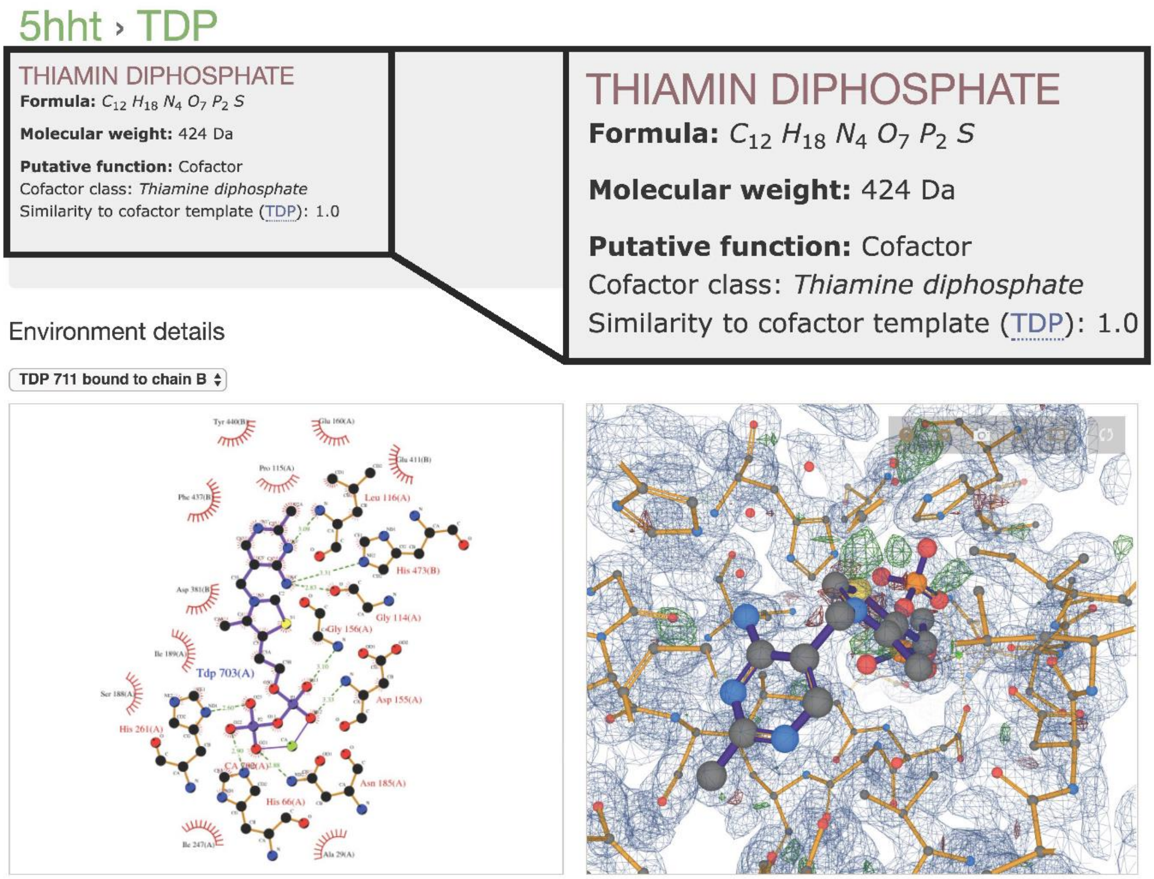

Supplement: btz115_Supplementary_Information [file btz115_supplementary_information.pdf]
